# Pck2 association with the plasma membrane and efficient response of the cell integrity pathway require regulation of PI4P homeostasis by exomer

**DOI:** 10.1098/rsob.240101

**Published:** 2024-11-13

**Authors:** Esteban Moscoso-Romero, Sandra Moro, Alicia Duque, Francisco Yanguas, M.-Henar Valdivieso

**Affiliations:** ^1^ Departamento de Microbiología y Genética, Universidad de Salamanca, Salamanca 37007, Spain; ^2^ Instituto de Biología Funcional y Genómica (IBFG), Consejo Superior de Investigaciones Científicas (CSIC), Calle Zacarías González 2, Salamanca 37007, Spain; ^3^ Department of Biosciences, University of Oslo, Oslo 0316, Norway

**Keywords:** cell integrity pathway, stress response, phosphatidylinositol 4-phosphate, phospholipids, trans-Golgi network, fission yeast

## Abstract

Exomer is a protein complex that facilitates trafficking between the Golgi and the plasma membrane (PM). *Schizosaccharomyces pombe* exomer is composed of Cfr1 and Bch1, and we have found that full activation of the cell integrity pathway (CIP) in response to osmotic stress requires exomer. In the wild-type, the CIP activators Rgf1 (Rho1 GEF) and Pck2 (PKC homologue) and the MEK kinase Mkh1 localize in the PM, internalize after osmotic shock and re-localize after adaptation. This re-localization is inefficient in exomer mutants. Overexpression of the PM-associated 1-phosphatidylinositol 4-kinase *stt4+*, and deletion of the *nem1+* phosphatase suppress the defects in Pck2 dynamics in exomer mutants, but not their defect in CIP activation, demonstrating that exomer regulates CIP in additional ways. Exomer mutants accumulate PI4P in the TGN, and increasing the expression of the Golgi-associated 1-phosphatidylinositol 4-kinase *pik1+* suppresses their defect in Pck2 dynamics. These findings suggest that efficient PI4P transport from the Golgi to the PM requires exomer. Mutants lacking clathrin adaptors are defective in CIP activation, but not in Pck2 dynamics or in PI4P accumulation in the Golgi. Hence, traffic from the Golgi regulates CIP activation, and exomer participates in this regulation through an exclusive mechanism.

## 1. Introduction

Vesicle trafficking mediates bidirectional transport of proteins between internal organelles and the cell surface, a phenomenon that allows communication between the cell interior and exterior. This transport requires the participation of protein coats and adaptors [1–8]. The Golgi plays a fundamental role in vesicle trafficking. In particular, the trans-Golgi network (TGN) and early endosomes, which in yeast can be considered equivalent functional compartments [9], serve as a classification centre that connects the secretory and endocytic routes. In the TGN, clathrin associates with diverse adaptors (AP complexes, GGAs and epsins) to coat vesicles destined for the plasma membrane (PM) and the vacuole [2,3,10].

Exomer is a hetero-tetrameric complex, initially described in *Saccharomyces cerevisiae* that populates the TGN. It is composed of two subunits of Chs5 and two ChAPs (Chs5 and Arf1-binding proteins: Bch1, Bch2, Chs6 and Bud7). It is essential for the delivery of the chitin synthase Chs3 from the TGN to the PM, such that in exomer mutants Chs3 stalls in the TGN, leading to a chitin synthesis defect. For this reason, and because of structural similarities with the clathrin adaptors and the lack of interaction with clathrin, exomer is considered a clathrin-independent cargo adaptor. Other exomer cargoes, which share with Chs3 the presence of several transmembrane helixes and a polarized distribution, were identified later [11–23]. Nevertheless, the absence of exomer leads to additional defects that cannot be ascribed to the retention of bona fide cargoes in the TGN. Thus, exomer mutants are sensitive to alkali metal cations, and this sensitivity has been related to a non-polarized distribution of the ion pump Ena1 in the PM [12,24]. This result underscores the role of exomer in polarized secretion [25] and demonstrates that it is not always essential for exiting the TGN. All fungi have exomer components, but its composition is variable. It always bears a Chs5 homologue, and can have 1, 2 or 4 ChAPs, which arose from the ancient ChAP Bch1 through diverse duplication events. The function of exomer as a Chs3 adaptor was a late acquisition that arose concomitant with the presence of chitin in the fungal cell wall [12,26,27]. *Schizosaccharomyces pombe* has no detectable amounts of chitin and bears the simplest functional exomer, composed of the Chs5 homologue Cfr1 and one ChAP (Bch1) [28–30]. *S. pombe* exomer mutants exhibit mild defects in multiple processes: cell fusion during mating, the distribution of some lipids, membrane polarization, K^+^ and Ca^2+^ homeostasis, polarized distribution of the K^+^-extrusion pump Cta3 and the Ca^2+^ channel Pkd2, growth in the presence of these cations and septum synthesis in the presence of KCl [29,31,32]. Thus, exomer plays a role in polarized secretion and in the maintenance of ion homeostasis in both organisms.

Cells are constantly subjected to variations in intra- and extracellular conditions, something that is particularly challenging for unicellular organisms. Consequently, they have developed mechanisms to ensure cell integrity; these mechanisms rely on signalling pathways that sense the stimuli and fire responses to promote adaptation and survival. Typically, the pathways comprise cell surface sensors that detect the stimuli and transmit a signal to a mitogen-activated protein kinase (MAPK) cascade that, in turn, activates the effectors. No sensors have been identified for the *S. pombe* cell integrity pathway (CIP), which is composed of the MAPKs Mkh1, Pek1/Skh1/Mkk1 and Pmk1/Spm1. These kinases are regulated by upstream activators, which include the Rho1 and Rho2 GTPases and the Pck1 and Pck2 PKC homologues. The CIP responds to high temperature, cell wall damage, and saline and osmotic stress, and its dysfunction results in defects in cell integrity and in cytokinesis [33–35].

Vesicle trafficking and signalling pathways cross-regulate, contributing to cell adaptation and integrity. Vesicles mediate the interchange of proteins between organelles, but also the interchange of lipids, some of which give identity to different membranes and ensure the correct directionality of transport [36–38]. Additionally, lipids influence signalling pathways because they interact with membrane-associated sensors and enzymes. This interaction determines their localization and impact on their function by providing an adequate environment [39]. In turn, regulatory proteins of signalling pathways, such as GTPases, can modify and regulate proteins that participate in lipid homeostasis and in trafficking. In particular, the Golgi and endosomes are considered signalling platforms because they accommodate components of diverse signalling pathways and participate in their activity, actions that modulate trafficking according to the cell demand [40,41].

*S. pombe* exomer mutants are sensitive to ions. Hence, in the current study we examined the role of exomer in stress response. We found that the exomer mutants were defective in the CIP response to osmotic stress because of reduced association of Rgf1, Pck2 and Mkh1 with the PM after the initial shock. The defect in Pck2 was suppressed by increasing the availability of phospholipids (PLs), in particular by increasing the synthesis of PI4P in the PM or in the Golgi. Additionally, exomer mutants accumulated this lipid in the TGN, a finding that point to a role for exomer in its delivery to the PM. We also found that mutants that lack other adaptors that operate in the Golgi exhibited defects in CIP activation and Pck2 distribution. However, only exomer mutants were defective in Pck2 re-association with the PM after the initial osmotic shock, disproving the hypothesis that this phenotype is a consequence of abnormal trafficking from the TGN.

## 2. Results

### 2.1. Exomer mutants are defective in CIP activation in response to osmotic stress

**Figure 1.**
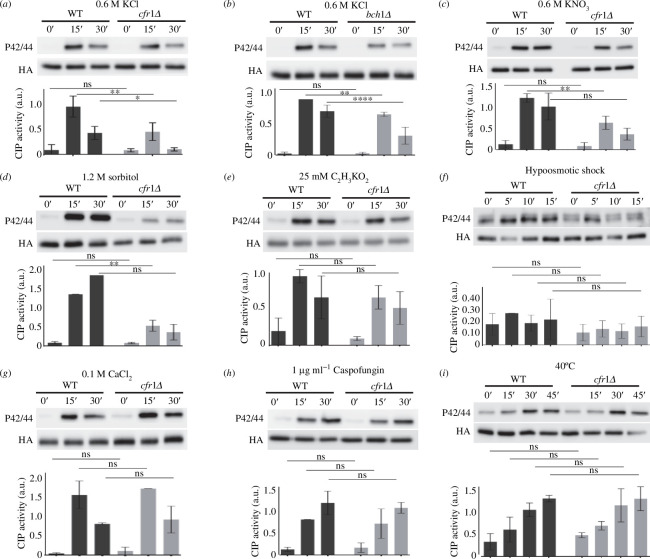
Activation of the cell integrity pathway (CIP) in response to osmotic shock is defective in exomer mutants. (*a*) Cells from the wild-type control (WT) and the exomer mutant *cfr1Δ* were exposed to 0.6 M of KCl for the indicated times (minutes). CIP activation was analysed in purified Pmk1-HA:6His samples by western blot using anti-p42/44 (phosphorylated Pmk1) and anti-HA (total Pmk1) antibodies. (*b*) The same as in (*a*), but the exomer mutant was *bch1Δ*. (*c*) CIP activation in cells treated with 0.6 M of KNO_3_. (*d*) The indicated strains were collected by filtration, transferred from YES to YES with 1.2 M of sorbitol, incubated for the indicated times, and analysed for CIP activation. (*e*) CIP activation in cells treated with 25 mM of C_2_H_3_KO_2_. (*f*) Cells growing in YES with 0.8 M sorbitol were transferred to YES, incubated for the indicated times, and analysed for CIP activation. (*g*) CIP activation in cells treated with 0.1 M of CaCl_2_. (*h*) CIP activation in cells treated with 1 µg/ml of caspofungin. (*i*) CIP activation in cells incubated at 40°C for the indicated times. All the analyses were performed a minimum of three times. A representative blot is shown. The bar graphs depicted below the blots represent the CIP activity, calculated as the ratio between the p42/44 (phosphorylated Pmk1) and HA (total Pmk1) signals. They show the mean and standard deviation. a.u., arbitrary units. The Šidák correction was used after ANOVA to determine the statistical significance of the differences. ns, non-significant; *p < 0.05; **p < 0.01; ****p < 0.0001.

The fact that mutants lacking exomer are sensitive to potassium chloride (KCl) and exhibit defects in septation in the presence of KCl [29] prompted us to analyse their CIP response. We found that the extent of the response in *cfr1Δ* cells treated with 0.6 M of KCl was about 50% that of the wild-type control (WT; Figure 1*a*). We obtained a similar result for *bch1Δ* (figure 1*b*). To determine whether exomer mutants were defective in the response to chloride, potassium or osmotic shock, we analysed CIP activation in the WT and *cfr1Δ* cells treated with different compounds. The mutant was defective in the CIP response to 0.6 M of potassium nitrate (KNO_3_; Figure 1*c*) and to 1.2 M of sorbitol (figure 1*d*), but not to 25 mM of potassium acetate (C_2_H_3_KO_2_; Figure 1*e*), a concentration that was low enough to allow efficient growth of the WT. These results suggested that exomer mutants were defective in the response to osmotic shock. Next, we analysed the CIP response to hypoosmotic shock by transferring the cells from yeast extract with supplements (YES) medium supplemented with 0.8 M of sorbitol to YES medium (figure 1*f*). Under this condition, the range of the response was lower than that observed with other stimuli, which increased the variability of the results; consequently, the differences between the WT and the mutant were not significant. Nevertheless, the signal for the mutant was always lower than that for the WT, suggesting that exomer might be required for effective CIP activation in response to hypotonic shock. Finally, we analysed the response to some stressors that did not involve changes in osmolarity. We analysed the response to 0.1 M of CaCl_2_ because exomer mutants are defective in calcium homeostasis [32]. We also analysed the response to 1 µg ml^−1^ of caspofungin because exomer mutants have a mild defect in cell wall composition, and cell wall damage is a typical CIP activator [29,33,42,43]. As an additional source of stress, we analysed the CIP response to 40°C. We found that CIP activation was similar in the WT and *cfr1Δ* cells in response to these stressors (figure 1*g–i*).

Next, we wanted to analyse whether the defect in the CIP response was specific or indirect, since CIP crosstalks with other signalling pathways. There is a functional relationship between CIP, calcineurin and calcium uptake [33,44–46], and exomer mutants have high calcineurin activity and are defective in calcium homeostasis [32]. In agreement, we investigated whether the reduced CIP activation in *cfr1Δ* was related to those defects. To answer this question, we deleted *ppb1+* (the gene that codes for the calcineurin catalytic subunit) and measured CIP activation in response to KCl. We found that the level of CIP activation was significantly greater in the *ppb1Δ* background strains than in the *ppb1+* control strains (compare *ppb1Δ* with the WT, and *cfr1Δ ppb1Δ* with *cfr1Δ* in electronic supplementary material, figure S1A). Nevertheless, the level of Pmk1 phosphorylation was still lower in *cfr1Δ ppb1Δ* than in *ppb1Δ*, a result that showed that the enhanced calcineurin activity exhibited by exomer mutants was not responsible for their defect in CIP activation.

The expression of phosphatases that dephosphorylate Pmk1 and therefore downregulate the CIP depends on the stress-activated protein kinase (SAPK) Sty1 [47]. Accordingly, we investigated the possibility that the reduced CIP activity was the consequence of Sty1 hyperactivation and/or the accumulation of Pmp1, the most specific Pmk1 phosphatase [33,46,48,49]. As shown in electronic supplementary material, figure S1B, the level of Sty1 phosphorylation in response to KCl was similar in the WT and *cfr1Δ* strains. Moreover, the level of Pmp1 was not greater in the *cfr1Δ* or the *bch1Δ* exomer mutants than in the WT (electronic supplementary material, figure S1C).

There is crosstalk between CIP and TORC2, and mutants defective in TORC2 components and regulators are sensitive to KCl [50–53]. Consequently, we investigated whether the reduced CIP activation depended on TORC2 by analysing the CIP response to KCl in the absence of the TORC2 kinase Tor1. We found that Pmk1 phosphorylation was less efficient in *tor1Δ cfr1Δ* than in *tor1Δ* (electronic supplementary material, figure S1D), a result that disproved the hypothesis.

In summary, all these results show that exomer mutants are defective in the CIP response to hyperosmotic stresses and that this defect is specific, and not a consequence of deregulation of the calcineurin, SAPK or TORC2 signalling pathways.

### 2.2. Exomer is not a component of the CIP

**Figure 2.**
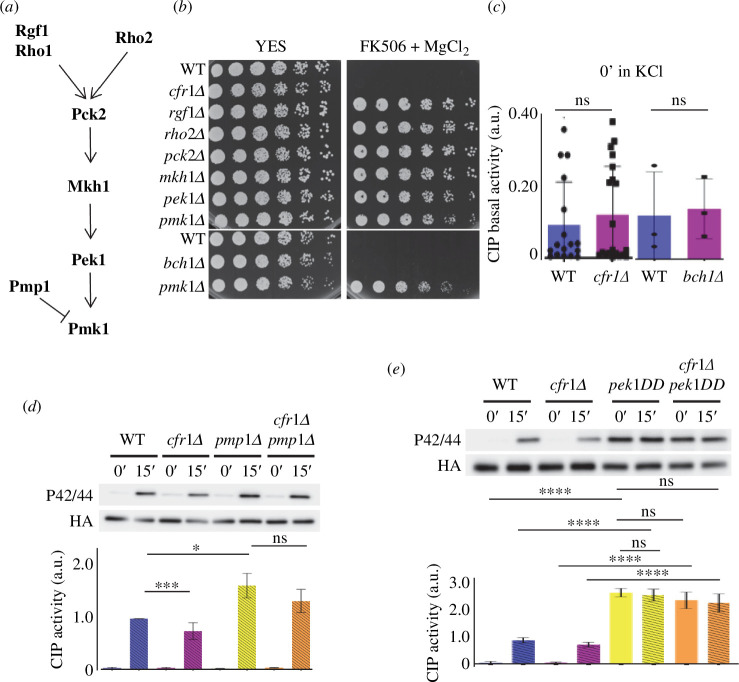
Exomer is not a component of the cell integrity pathway (CIP). (*a*) Schematic representation of the order of action of the CIP components analysed in this work. (*b*) Analysis of the viable in immunosuppressant and chloride (VIC) phenotype in the indicated strains. 3 × 10^4^ cells and serial 1:4 dilutions were plated on YES and YES with 1 μg ml^−1^ of FK506 and 0.2 M MgCl_2_ plates. The plates were incubated at 32°C for 2 days. (*c*) CIP activity in the wild-type control (WT) and the *cfr1Δ* or the *bch1Δ* mutants growing in YES medium. The activation was analysed in purified Pmk1-HA:6His samples by western blot using anti-p42/44 (phosphorylated Pmk1) and anti-HA (total Pmk1) antibodies. (*d*) The same as in (*c*), but using the WT and *pmp1Δ*, *cfr1Δ* and *cfr1Δ pmp1Δ* strains treated with 0.6 M of KCl for the indicated times. (*e*) The same as in (*d*), but using the WT, *cfr1Δ*, *pek1-S234D,T238D* (*pek1DD*) and *cfr1Δ pek1DD* strains. In (*c*–*e*), the bar graphs depicted below the blots represent the CIP activity, calculated as the ratio between the p42/44 (phosphorylated Pmk1) and HA (total Pmk1) signals. They show the mean and standard deviation. a.u., arbitrary units. The *t*‐test and Tukey correction after ANOVA were used to determine the statistical significance of the differences in (*c*) and in (*d,e*), respectively. ns, non-significant; *p < 0.05; ***p < 0.001; ****p < 0.0001.

The fact that the defect in CIP activation in *cfr1Δ* and in *bch1Δ* was partial suggests that exomer is not an essential component of this pathway; nevertheless, we investigated this possibility in more detail. Mutants in the essential components of the pathway (depicted in figure 2*a*) exhibit the characteristic VIC phenotype (viable in the presence of immunosuppressant and chloride ion [33,45,46]). Consequently, we compared the growth of *cfr1Δ* and *bch1Δ* on YES plates supplemented with magnesium chloride and FK506 with that of the WT and CIP mutants. As shown in figure 2*b*, the WT and the exomer mutants were sensitive to this condition, while the rest of the mutants grew efficiently. In agreement, the basal CIP activation, measured in cells growing in the absence of KCl, was similar in the WT and exomer mutants (figure 2*c*).

Next, we used two genetic approaches to determine whether exomer was essential for maximum CIP activation when the pathway was activated constitutively. First, we compared CIP activity in the WT and *cfr1Δ* strains lacking the Pmp1 phosphatase [46]. As expected, the extent of Pmk1 phosphorylation, after 15 min in 0.6 M of KCl, was significantly greater in *pmp1Δ* than in the WT (figure 2*d*). Under these conditions, Pmk1 phosphorylation was similar in *pmp1Δ* and *cfr1Δ pmp1Δ* strains, a result that showed suppression of the *cfr1Δ* phenotype. Second, we used strains bearing an integrated version of the *pek1-S234D,T238D* phosphomimetic mutant [54] (hereafter *pek1DD*), under the control of the strong *act1+* promoter (see Materials and methods). In this background, the level of Pmk1 phosphorylation was enhanced with respect to that of the control *pek1+* strains in the absence of stress (figure 2*e*). A 15 min treatment with 0.6 M of KCl did not enhance Pmk1 phosphorylation further, in agreement with constitutive activation. Additionally, there were no differences in the level of activation between the *cfr1+ pek1DD* and the *cfr1Δ pek1DD* strains grown in YES or in YES with KCl, showing that Pmk1 hyperactivation suppressed the defect in CIP activation of exomer mutants.

Together, these results confirm that exomer is not an essential CIP component and strongly suggest that exomer regulates the pathway upstream of Pmk1.

### 2.3. The distribution of CIP regulators is altered in exomer mutants exposed to KCl

**Figure 3.**
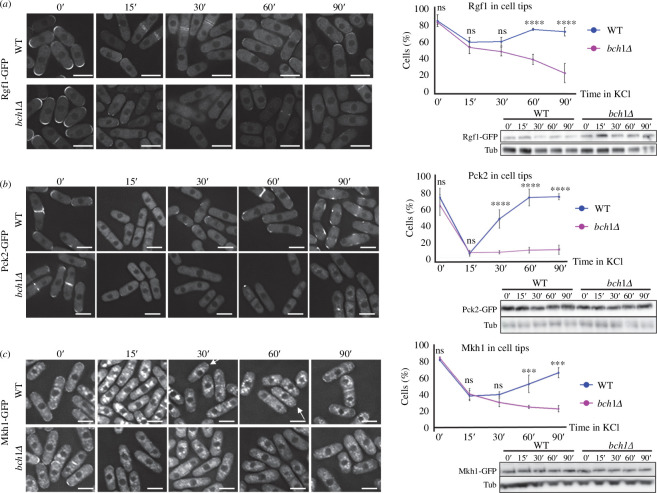
The distribution of the cell integrity pathway ( CIP ) regulators is altered in exomer mutants exposed to KCl. (*a*) Left panel : wild-type (WT) and *bch1Δ* cells, expressing Rgf1-GFP and treated with 0.6 M of KCl, were photographed after the indicated times (minutes) with a DeltaVision system. The images are AVG projections. Upper right panel : the percentage of cells exhibiting Rgf1-GFP at the cell surface at the indicated time points. Cells with fluorescent signal in the cell poles and cell sides, but not in the cell equator and septa were scored ( Materials and methods). Lower right panel : the cell extracts from the same strains treated with 0.6 M of KCl were analysed by western blot using anti-GFP and anti-tubulin (Tub; loading control) antibodies. (* b *) The same as in (*a* ), but the cells expressed Pck2-GFP. (*c*) The same as in ( *a* ), but the cells expressed Mkh1-GFP. The arrows denote examples of cells with Mkh1-GFP at the poles. The Šidák correction was used after ANOVA to determine the statistical significance of the difference between the value for the WT and *bch1Δ* strains treated for the same time is indicated. ns, non-significant; *** *p* < 0.001; **** *p* < 0.0001. Scale bar, 10 µm.

To gain information about the role of exomer in the regulation of the CIP, we analysed the localization of the main CIP regulators and components. The Wsc1 and Mtl2 cell surface mechanosensors regulate Rho1 in response to stress, but do not act in CIP signalling [55]. Nevertheless, we analysed their distribution and found that it was similar in the WT and *cfr1Δ* strains, in both YES and YES with 0.6 M of KCl for up to 1 h (electronic supplementary material, figure S2). Rgf1 is a Rho1-GEF that activates CIP, and its presence at the cell surface of the cell poles is lost after adding 1 M of KCl [43,56]. We quantified the percentage of WT and *bch1Δ* cells exhibiting Rgf1-GFP at the cell surface at different incubation times in 0.6 M of KCl following the criteria explained in Materials and methods and in the electronic supplementary material, figure S3. In the WT, this percentage decreased 15 minafter KCl addition, remained low at 30 min, increased from this time point reaching a value similar to that of the 0 min at 60 min, and remaining high at 90 min (figure 3*a*). In the mutant, the percentage of cells with Rgf1 at the surface was similar to that of the WT at the early time points (0, 15 and 30 min), but continued to decrease during the experiment. Western blot analyses showed that the lack of Rgf1 at the cell surface was not the consequence of protein degradation. This result indicated that Rgf1 association with the PM was reduced in response to osmotic shock, and that this reduction lasted longer in the absence of exomer.

Rho2, the GTPase that transmits the stress signal produced by osmotic changes to Pmk1 [42], is uniformly distributed in the PM under basal conditions. Under osmotic shock it is observed in a series of clusters that co-localize with phosphatidylinositol 4,5-bisphosphate (PI(4,5)P2), and this association is important for CIP activation [56]. In exomer mutants, the percentage of cells exhibiting a uniform distribution of PI(4,5)P2 at the cell periphery is altered [32]. When we quantified the percentage of WT cells that exhibited this phosphoinositide (PI) in clusters after adding 0.6 M of KCl, we found that the values followed a saw-tooth pattern along the experiment, while *cfr1Δ* cells showed less variability (electronic supplementary material, figure S4A). This difference prompted us to analyse Rho2 distribution in response to KCl. We did not find differences between the WT and mutant strains regarding the timing of Rho2 clustering, the number of cells with clusters, the amount of Rho2 protein or the intensity of GFP-Rho2 fluorescence on the cell surface (in and out of the clusters). Moreover, mCherry-Rho2 co-localized with the PI(4,5)P2-binding probe GFP-h\GAP1IP4BP(PH) in both strains (electronic supplementary material, figure S4B–E). These results indicated that the exomer defect in CIP signalling was not due to alterations in Rho2.

The PKC orthologue Pck2 is an upstream regulator of the CIP—whose presence in the cell poles is required for activation of the pathway—and it localizes to the cytoplasm after osmotic shock [56–58]. When we quantified the percentage of cells with Pck2 at the cell surface, the results indicated that the osmotic shock had a greater effect on the distribution of this protein than that observed for Rgf1. In the WT strain, the percentage of cells with Pck2 at the surface decreased 15 min after the addition of 0.6 M of KCl to values lower than those observed for Rgf1 (compare the graphs in figure 3*a,b*). This value increased at 30 min, and continued increasing at later time points. The percentage of *bch1Δ* cells with Pck2 at the cell surface was similar to that of the WT at 0 min and 15 min, and remained low for the rest of the experiment. Western blotting showed that the amount of Pck2-GFP was similar in both strains and did not vary after adding KCl (figure 3*b*). The dynamics of Pck2 re-association with the cell surface were also altered in *cfr1Δ* (electronic supplementary material, figure S2B).

Mkh1, the CIP mitogen-activated protein kinase kinase kinase (MAPKKK), localizes in the cell poles in a Pck2-dependent manner, and this localization is required for proper CIP signalling [58,59]. Consequently, we quantified the presence of this kinase in the cell surface after osmotic shock (figure 3*c*). We found that most of the WT cells had Mkh1-GFP fluorescence in the poles. This percentage decreased 15 min after the addition of 0.6 M of KCl, and then increased until the end of the experiment (90 min). In the case of *bch1Δ*, the values were similar to those of the WT at 0 min and 30 min, and continued decreasing until the end of the experiment. The amount of Mkh1-GFP was similar in both strains at all the time points (figure 3*c*).

We did not analyse the distribution of Pek1-GFP in the presence of KCl because its fluorescence was too low to follow along the experiment. Regarding Pmk1-GFP, this kinase localizes in the cytoplasm, nucleus, septum and mitotic spindle under both basal and stress conditions [60,61]. In general, the localization of Pmk1 in the WT and exomer mutants was similar in YES and YES with 0.6 M of KCl (electronic supplementary material, figure S5). We only found a significant difference in the percentage of cells that exhibited Pmk1-GFP in the septal area, which was higher in the mutant after 60 min in KCl. This difference, which we did not detect at earlier or later time points, might be related to the facts that Pmk1 participates in a vigilance mechanism for acto-myosin ring constriction under stress conditions, and that exomer mutants produce abnormal septa when incubated in KCl [29,34]. According to western blotting, the amount of Pmk1 was similar in both strains along the experiment (electronic supplementary material, figure S5).

In summary, the localization of the upstream CIP regulators Rgf1 and Pck2, and the MAPKKK Mkh1 at the cell surface is lower in the absence of exomer than in the WT cells in the presence of KCl. Moreover, this reduction is not due to a reduced amount of protein.

### 2.4. In exomer mutants, the defect in CIP activation correlates with defects in Pck2 localization

**Figure 4.**
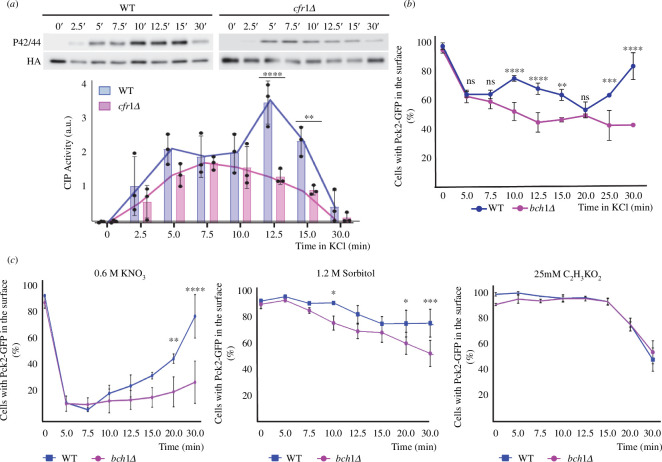
Inefficient cell integrity pathway ( CIP ) activation correlates with defects in the localization of Pck2. ( *a* ) Cells from the wild-type control (WT) and the exomer mutant *cfr1Δ* were exposed to 0.6 M of KCl for the indicated times (minutes). CIP activation was analysed in purified Pmk1-HA:6His samples by western blot using anti-p42/44 (phosphorylated Pmk1) and anti-HA (total Pmk1) antibodies. The bar graphs depicted below the blots represent the CIP activity, calculated as the ratio between the p42/44 (phosphorylated Pmk1) and HA (total Pmk1) signals. They show the mean and standard deviation. a.u., arbitrary units. Line graphs for the same values have been depicted to facilitate the comparison of the CIP activation dynamics in both strains. (* b *) Percentage of cells incubated in YES with 0.6 M of KCl for the indicated times that exhibited Pck2-GFP at the cell poles and cell sides, but not in the cell equator and septa ( Materials and methods). (* c *) The same as in ( *b* ), but the cells were exposed to 0.6 M of KNO_3_ (left panel), 1.2 M of sorbitol (central panel) or 25 mM of C_2_H_3_KO_2_ (right panel). The Šidák correction was used after ANOVA to determine the statistical significance of the difference between the value for the WT and the mutant strains treated for the same time. ns, non-significant; * *p* < 0.05¸** *p* < 0.01; *** *p* < 0.001; **** *p* < 0.0001.

We were intrigued by the fact that even though exomer mutants had a defect in CIP activation 15 min after osmotic shock (figures 1 and 2; electronic supplementary material, figure S1), at this time the percentage of WT and mutant cells with CIP components at their surface was similar (figure 3). To gain more information about the relationship between both processes, we analysed CIP activation and the distribution of CIP regulators at short time intervals. We found that in the WT, CIP activation was detectable 2.5 min after the addition of KCl, and then followed a biphasic pattern, with a small Pmk1 phosphorylation peak at 5 min, and a larger peak at 12.5 min (figure 4*a*). From that point, the level of Pmk1 phosphorylation decreased, reaching its minimum value at 30 min. In the *cfr1Δ* strain, the level of Pmk1 phosphorylation did not follow a biphasic pattern. Instead, it increased slowly and progressively from the 2.5 min time point, reaching its maximum at 7.5 min, with a value similar to that of the WT at this time point. Later, the level of Pmk1 phosphorylation decreased slowly, reaching its minimum value at 30 min. The difference between the values for both strains was significant at 12.5 and 15 min.

Next, we determined the percentage of cells exhibiting a CIP regulator at the cell surface at the same time points. We chose Pck2 because the fluorescence of Pck2-GFP was stronger than that of Rgf1-GFP and Mkh1-GFP, making it easier to identify the cells that exhibited this protein in their surface (figure 3). Additionally, Pck2-GFP underwent the strongest changes in its distribution (figure 3), and therefore it allowed us to observe more subtle differences between the time points. Moreover, Pck2 channels Rgf1 signalling to Mkh1, and it is necessary for proper Mkh1 localization and activation [43,58]. Thus, we reasoned that analysis of Pck2 would provide information about the behaviour of the other regulators. Additionally, for the experiment shown in figure 4*b* we used a confocal Dragonfly system, instead of a DeltaVision system, because of its enhanced sensitivity. Consequently, the values in figures 3*b* and 4*b* cannot be compared directly. In the WT, the percentage of cells with Pck2 at the cell surface decreased from the value obtained in the absence of KCl 5 min after adding KCl to the culture, and remained low after an additional 2.5 min incubation. At 10 min, it experienced a mild increase and then decreased, reaching its minimum value at 20 min, which produced a small peak at 10 min. From this time point, the percentage of the cells that exhibited Pck2 at the cell poles increased until the end of the experiment (figure 4*b*). For *cfr1Δ* and for *bch1Δ*, the values did not follow this biphasic pattern (figure 4*b*; electronic supplementary material, figure S2C). Thus, the strains exhibited different dynamics regarding Pck2 association with the cell surface from early time points.

The results showed that both activation of the CIP pathway and localization of Pck2 were defective in the absence of exomer soon after the osmotic shock. Next, we analysed the presence of Pck2 at the cell surface of WT and mutant cells treated with other stressors. We found that after exposure to 0.6 M of KNO_3_ or 1.2 M of sorbitol, fewer *bch1Δ* cells had this protein at the surface compared with WT cells (figure 4*c*, left and central panels). On the contrary, we did not find a significant difference between the strains when we added 25 mM of C_2_H_3_KO_2_ to the cultures (figure 4*c*, right panel). In summary, these results reveal a correlation between defects in the dynamics of Pck2 association with the cell surface and defects in CIP activation in exomer mutants. This is in agreement with the fact that Pck2 transduces the response to changes in osmolarity [33,42].

### 2.5. Increasing the availability of PI4P and other phospholipids (PLs) in the PM suppresses the defect in Pck2 distribution of exomer mutants

**Figure 5.**
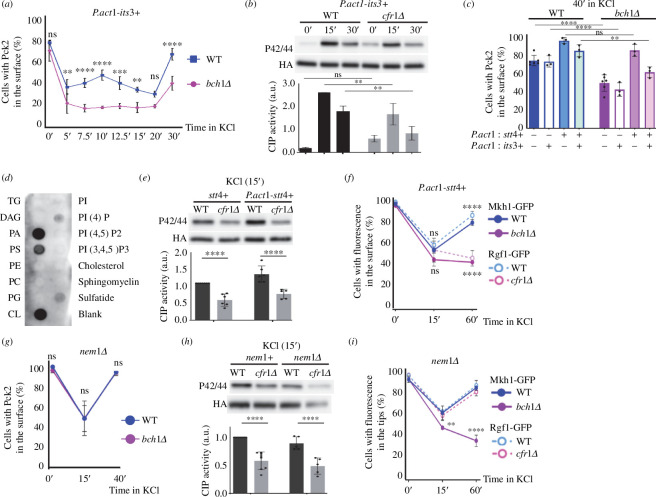
Relationship between plasma membrane lipids, Pck2 localization and cell integrity pathway ( CIP ) activation. ( *a* ) Cells from the control (WT) and *bch1Δ* strains that bear Pck2-GFP and overexpress *its3+* were treated with 0.6 M of KCl for the indicated times (minutes), and photographed. The percentage of cells exhibiting fluorescence in the cell poles and cell sides, but not in the cell equator and septa ( Materials and methods) was scored from the images. (* b *) CIP activation in the control (WT) and *cfr1Δ* cells that overexpress *its3+* exposed to 0.6 M of KCl for the indicated times (minutes). (* c *) Percentage of WT and *bch1Δ* cells bearing Pck2-GFP that exhibit GFP fluorescence in the cell surface, estimated as in ( *a* ), after 40 min in the presence of 0.6 M of KCl. The cells expressed the indicated genes from the *act1+* promoter. (* d *) Protein–lipid overlay assay to assess Pck2 binding to membrane lipids. TG, triglyceride; DAG, diacylglycerol; PA, phosphatidic acid; PS, phosphatidylserine; PE, phosphatidylethanolamine; PC, phosphatidylcholine; PG, phosphatidylglycerol; PI, phosphatidylinositol; PI4P, phosphatidylinositol 4-phosphate; PI(4,5)P2, phosphatidylinositol 4,5-bisphosphate; PI(3,4,5), phosphatidylinositol 3,4,5-trisphosphate; sulfatide, 3-sulfogalactosylceramide. (* e *) The same as in ( *b* ), but the cells expressed Stt4 form its native promoter (*stt4+*) or from the *act1+* promoter (*P.act1-stt4+*). (* f *) The same as in ( *a* ), but the WT and mutant cells (blue and magenta lines, respectively) expressed *stt4+* from the *act1+* promoter, and carried Mkh1-GFP (solid lines) or Rgf1-GFP (dashed lines). (* g *) The same as in ( *a* ), but the WT and *cfr1Δ* cells contained a *nem1Δ* deletion. (* h *) CIP activation in *nem1+* and *nem1Δ* cells was analysed as in (*b*). ( *i* ) Percentage of WT and exomer mutant cells (blue and magenta lines, respectively) deleted for *nem1+* that expressed Mkh1-GFP (solid lines) or Rgf1-GFP (dashed lines) and exhibited fluorescence in the cell surface, estimated as in ( *a* ). In ( *b* ), *( e* ) and ( *h* ), CIP activation was analysed in purified Pmk1-HA:6His samples by western blot using anti-p42/44 (phosphorylated Pmk1) and anti-HA (total Pmk1) antibodies. The bar graphs represent the ratio between the intensity of the p42/44 band and the corresponding HA band. a.u., arbitrary units. The statistical significance of the differences between the values for the WT and mutant strains treated for the same time is indicated where appropriate. The Šidák correction was used after ANOVA in ( *a –c* ), *( e* ) and ( *g, h* ). The Tukey correction was used in ( *f* ) and ( *i* ). In ( *e* ) and ( *h* ), the asterisks denote the significance of the difference between the values for each strain at 0 and 15 min of exposure to KCl. ns, non-significant; * *p* < 0.05¸** *p* < 0.01; *** *p* < 0.001; **** *p* < 0.0001.

PKC regulation and CIP activation require PI(4,5)P2 [56,62]. Exomer mutants exhibit an alteration in the distribution of this lipid in response to KCl [32] (electronic supplementary material, figure S4A) and, according to the fluorescence of the GFP-h\GAP1IP4BP(PH) probe, its amount was reduced in the mutants (electronic supplementary material, figure S6A). Consequently, we investigated whether increasing its amount would suppress the defects in Pck2 distribution and CIP activation. We generated a strain that expressed the 1-phosphatidylinositol 4-phosphate 5-kinase *its3+* from the strong *act1+* promoter, which would allow an enhanced constitutive expression (PomBase; https://www.pombase.org/) [63]. This construct increased the fluorescence of GFP-h\GAP1IP4BP(PH) (electronic supplementary material, figure S6A), produced swollen cells (electronic supplementary material, figures S6A and S7A), induced the formation of PM invaginations and bubbles (electronic supplementary material, figure S6B), and increased CIP activation (electronic supplementary material, figure S6C), phenotypes that were expected from enhanced *its3+* expression [56,64]. Additionally, this construct suppressed the defect in PI(4,5)P2 distribution of exomer mutants (electronic supplementary material, figure S6D). Nonetheless, it did not suppress the defect in Pck2 association with the PM or CIP activation (figure 5*a,b*).

We hypothesized that suppression of these defects might require an amount of PI(4,5)P2 that might not be reached in these strains because of a shortage in PI4P, the Its3 substrate. To address this question, we constructed strains that expressed both *its3+* and the PM-associated 1-phosphatidylinositol 4-kinase *stt4+* from the *act1+* promoter. As a control, we generated strains that only expressed *stt4+* from the strong promoter. We analysed Pck2 behaviour in these strains by quantifying the percentage of cells with Pck2 in the cell surface at 0, 15 and 40 min after adding 0.6 M of KCl (figure 5*c*; electronic supplementary material, figure S7B). At 40 min, this percentage was higher in the *bch1Δ* strain that overexpressed *its3+* and *stt4+* than in the strain that only overexpressed *its3+* (figure 5*c*), data that support the hypothesis of PI4P shortage. Surprisingly, in the strain that only overexpressed *stt4+*, there was total suppression of the *bch1Δ* defect. *stt4+* overexpression also suppressed the defect in Pck2 distribution in *cfr1Δ* cells (electronic supplementary material, figure S8A). These results strongly suggests that exomer mutants have a defect in PI4P and that although PI(4,5)P2 is involved in Pck2 association with the PM, PI4P plays a relevant role in this association. When we analysed the level of PI4P and PI(4,5)P2 in cells that expressed *stt4+* from the *act1+* promoter, we found no increase in the fluorescence of the PI4P-binding probe GFP-2xPH(Osh2) [65] (electronic supplementary material, figure S9A) but a significant increase in the fluorescence of the PI(4,5)P2- binding probe GFP-h\GAP1IP4BP(PH) (electronic supplementary material, figure S9B), which indicated that PI4P was being converted to PI(4,5)P2 very efficiently, as described in other organisms [66].

All these results strongly suggest that Pck2 association to the PM requires the presence of both, PI4P and PI(4,5)P2, and that exomer mutants have a shortage in PI4P.

To investigate whether Pck2 interacted with PI4P, we expressed it in bacteria and performed an overlay assay using membrane lipid strips (see Materials and methods). According to the results (figure 5*d*), Pck2 had some affinity for PI4P and for phosphatidylserine (PS). If the weak Pck2 interaction with PI4P detected in the lipid strip reflects *in vivo* binding, PI4P might contribute to Pck2 stabilization in the PM, which would explain the suppression of the mutant phenotype by *stt4+* overexpression (figure 5*c*). To address the possibility that a deficiency in PS might contribute to the defect in Pck2, we analysed its distribution using a lactC2-GFP probe [67]. The fluorescence intensity was enhanced, rather than reduced, in *cfr1Δ* cells at all the time points (electronic supplementary material, figure S9C). This finding argues against the idea of a defect in PS in the inner leaflet being responsible for the inefficient association of Pck2 with the PM.

Because we showed that the defect in Pck2 association with the PM correlated with defects in CIP activation (figure 4), we investigated whether *P.act1-stt4+*suppressed the CIP defect in *cfr1Δ*. We found that this was not the case (figure 5*e*). Analysis of Rgf1 and Mkh1 demonstrated that they did not re-localize to the PM in the exomer mutants after osmotic shock (figure 5*f*). These results show that the presence of Pck2 in the PM is necessary but not sufficient to activate CIP efficiently, and that PI4P does not stabilize either Rgf1 or Mkh1 at the PM.

Deletion of the lipin phosphatase is expected to increase the availability of PLs [68,69]. To complement the results described above, we analysed the effect of deleting the lipin regulator *nem1+* [70] on Pck2 association with the PM and in the CIP activation in the WT and exomer mutant. We found that the percentage of cells that exhibited Pck2-GFP fluorescence in the cell surface was similar in *nem1Δ* and in *nem1Δ bch1Δ* cells (denoted as WT and *bch1Δ*, respectively, in figure 5*g*). The fact that *nem1Δ* suppressed the defect in Pck2 re-association with the PM prompted us to investigate whether deleting *nem1^+^* + also suppress the CIP activation defect. As observed in figure 5*h*, this was not the case, since the level of CIP activation was lower in *nem1Δ cfr1Δ* than in the corresponding WT control (*nem1Δ*). Consequently, we analysed the dynamics of Rgf1 and Mkh1 re-association with the PM in the absence of exomer and Nem1. We found that the dynamics of Rgf1 re-association was similar in *nem1Δ* cells with and without exomer (figure 5*i*, WT and *cfr1Δ*, respectively), which showed suppression of the exomer mutant defect. On the contrary, Mkh1 re-association was not observed in the *nem1Δ* strain that lacked exomer (*bch1Δ* in figure 5*i*) after 40 min of incubation in KCl, a result that showed that this defect was not suppressed by eliminating Nem1. Thus, enhancing the expression of *stt4+* and deleting *nem1+* corrected the defect in Pck2, but not the defect in CIP activation, because other CIP regulators did not re-associate with the PM in the absence of exomer.

In summary, these results show that Pck2, Rgf1 and Mkh1 have different requirements for their association with the PM and that Pck2 association with the PM is necessary, but not sufficient, for full CIP activation. Additionally, they support the notion that exomer mutants have a defect in PM lipids that contributes significantly to the defect in Pck2 association with the cell surface, and therefore to the defect in CIP activation in response to osmotic stress.

### 2.6. Exomer mutants accumulate PI4P in the Golgi

**Figure 6.**
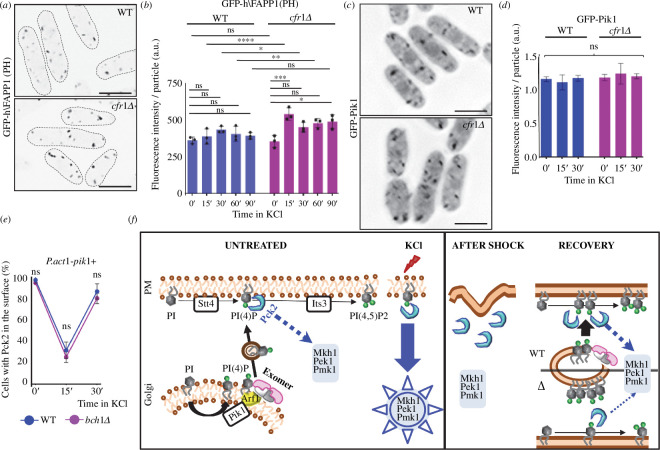
Phosphatidylinositol 4-phosphate (PI4P) homeostasis in the Golgi is altered in exomer mutants undergoing osmotic stress. ( *a* ) Micrographs of cells from the wild-type control (WT) and *cfr1Δ* strains bearing the GFP-h\FAPP1(PH) probe, which binds PI4P in the Golgi. The cells were treated with 0.6 M of KCl for 15 min . The images were captured with a Dragonfly spinning disk microscope and are average (AVG) projections. (* b *) The GFP fluorescence was quantified from photographs of the strains used in ( *a* ) treated with 0.6 M of KCl for the indicated times (minutes). (* c *) Micrographs of WT and *cfr1Δ* cells bearing GFP-Pik1. The images are AVG projections captured with a DeltaVision. (* d *) The GFP fluorescence was quantified from photographs of the strains used in (* c *) , treated with 0.6 M of KCl for the indicated times (minutes). (* e *) Control (WT) and *bch1Δ* cells that bear Pck2-GFP and express *pik1+* from the *P.act1+* promoter were treated with 0.6 M of KCl for the indicated times (minutes), and photographed. The percentage of cells exhibiting fluorescence in the cell poles and cell sides, but not in the cell equator and septa ( Materials and methods) were scored from the images. (* f *) Speculative model to explain the role of exomer in PI4P homeostasis and CIP regulation. Left panel, under basal conditions (UNTREATED), the 1-phosphatidylinositol 4-kinase Stt4 phosphorylates phosphatidylinositol (PI) and generates PI4P in the plasma membrane (PM). This PI4P is the substrate for the 1-phosphatidylinositol 4-phosphate 5-kinase Its3 that produces phosphatidylinositol 4,5-bisphosphate [PI(4,5)P2]. Pck2 binds to the PI4P and activates the CIP MAP kinase in response to stress (KCl). In the Golgi, 1-phosphatidylinositol 4-kinase Pik1 phosphorylates PI to generate PI4P. Exomer, which interacts with Arf1 and the Golgi membrane, would modulate the transfer of PI4P to the PM. Right panel, osmotic shock would alter the properties of the PM, and Pck2 would dissociate from the surface of cell poles (AFTER SHOCK). After the initial shock, the WT cells (WT) would adapt to the presence of KCl (RECOVERY); Its3 would synthesize PI(4,5)P2 from the PI4P generated in the PM by Stt4. Under these conditions, there would be enhanced transport of PI4P from the Golgi to the PM that would mediate Pck2 re-association with the PM. In the absence of exomer (Δ), this transport would be inefficient, which would result in PI4P accumulation in the Golgi, shortage of this lipid in the PM, and reduced Pck2 association with the PM and CIP signalling. In ( *a* ) and ( *c* ), scale bar, 10 µm. In ( *b* ) and ( *d* ), a.u., arbitrary units. In ( *b* ), *( d* ) and ( *e* ), the Šidák correction was used after ANOVA to determine the statistical significance of the differences. ns, non-significant; * *p* < 0.05; ** *p* < 0.01; *** *p* < 0.001; **** *p* < 0.0001.

Exomer acts in the TGN, and there is a pool of PI4P in the Golgi [29,71]. Consequently, we investigated how eliminating exomer would affect this pool. To do so, we used the GFP-h\FAPP1(PH) probe, which binds the Golgi PI4P [72,73]. As shown in figure 6*a* and electronic supplementary material, figure S8B, *cfr1Δ and bch1Δ* cells treated with 0.6 M of KCl for 15 min exhibited stronger fluorescence of the probe than WT cells. Quantification at various times showed that in WT cells there was a small reproducible, but non-significant, increase in the fluorescence, with a peak at 30 min. In *cfr1Δ*, the fluorescence increased significantly at 15 min and remained higher than in the WT cells and higher than in the mutant cells at 0 min along the experiment (up to 90 min; Figure 6*b*). *bch1Δ* cells also exhibited enhanced GFP-h\FAPP1(PH) fluorescence when they were incubated 15’ in KCl (electronic supplementary material, figure S8B). Observation of GFP-tagged Pik1 (the Golgi 1-phosphatidylinositol 4-kinase [74]) showed that this enzyme did not accumulate in the Golgi under the same conditions (figure 6*c,d*).

An explanation that reconciles the facts that the mutant had a defect in the PM PI4P pool and an excess in the Golgi pool is that exomer has a role in the transport of this lipid from the Golgi to the cell surface. To explore this possibility, we investigated whether increasing PI4P synthesis in the Golgi would bypass the requirement of exomer and thus mitigate the defect in Pck2 association with the PM. We expressed *pik1+* from the *act1+* promoter (see Materials and methods). The presence of swollen cells (electronic supplementary material, figures S7A, S8C and S9A,B,D) [75], and a significant increase in the fluorescence of the GFP-h\FAPP1(PH) probe (electronic supplementary material, figure S9D) confirmed increased *pik1+* expression. As shown in figure 6*e* and electronic supplementary material, figure S8C,D, under these conditions Pck2 re-associated with the PM efficiently 40 min after adding KCl. This result, together with an increase in the fluorescence of the GFP-2xPH(Osh2) probe (electronic supplementary material, figure S9A) confirms that PI4P overproduction in the Golgi bypasses the exomer requirement and replenishes the PI4P PM pool.

Together, these results support the hypothesis that exomer facilitates PI4P transport from the Golgi to the cell surface (see the model in figure 6*f*).

### 2.7. Efficient CIP activation and correct Pck2 distribution, but not Pck2 re-association with the PM after osmotic shock, depend on the clathrin adaptors that act in the TGN

**Figure 7.**
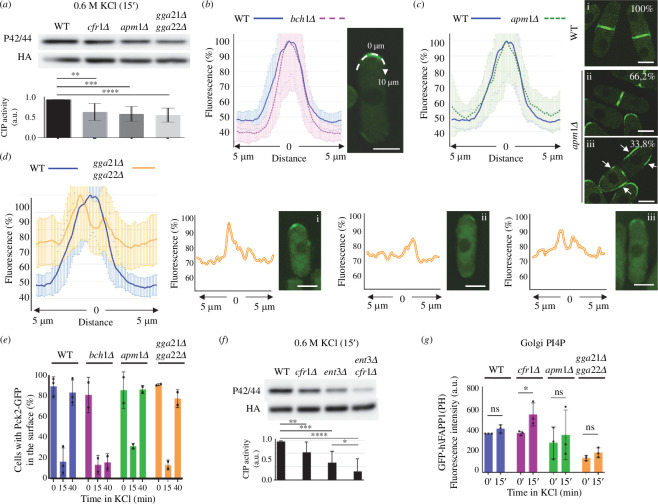
Role of Golgi clathrin adaptors in cell integrity pathway ( CIP ) activation, the regulation of Pck2 and phosphatidylinositol 4-phosphate (PI4P) homeostasis. ( *a* ) CIP activation in response to KCl was analysed in the wild-type (WT) strain and in strains lacking exomer (*cfr1Δ*), AP -1 (*apm1Δ*) and the GGA adaptors (*gga21Δ gga22Δ*). Western blotting was performed in purified Pmk1-HA:6His samples using anti-p42/44 (phosphorylated Pmk1) and anti-HA (total Pmk1) antibodies. (* b *) Line-scan analyses of the Pck2-GFP fluorescence intensity along the plasma membrane of cell poles in the WT and *bch1Δ* strains growing in YES. The graph in the left panel represents the mean and standard deviations of the plot profiles for the relative intensities of all the values obtained at different cell points from the cell tip toward the cell equator, as represented in the micrograph (right panel). (* c *) Left panel, the same as in ( *b* ), but the strains analysed were WT and *apm1Δ*. Right panel, micrographs from WT (i ) and *apm1Δ* (ii, iii) cells undergoing septation. The numbers indicate the percentage of cells exhibiting Pck2-GFP fluorescence in the septal area (i and ii ) or in the septal area and the cell sides (denoted by arrows in iii). (* d *) Left panel, the same as in ( *b* ), but the strains analysed were WT and *gga21Δ gga22Δ*. The three right panels are individual plot profiles of the line-scans of cells with different Pck2-GFP distributions, as shown in the corresponding micrographs (i–iii). The images were captured with a Dragonfly spinning disk confocal microscope and are average (AVG) projections. (* e *) Percentage of cells from the indicated strains that exhibited Pck2-GFP fluorescence at the cell poles and cell sides, but not in the cell equator and septa (§4 ). The cells were incubated in 0.6 M of KCl for the indicated times (minutes). The experiment was performed twice with similar results. (* f *) The same as in ( *a* ), but the strains lacked Cfr1, Ent3 or both proteins. (* g *) Intensity of the GFP fluorescence in the indicated strains that bear the GFP-h\FAPP1(PH) probe as an indicator of the level of PI4P in the Golgi. The cells were incubated with 0.6 M of KCl for the indicated times (minutes). The experiment was performed three times, and the statistical significance of the differences between the values for each strain at 0 and 15 min are indicated. In ( *a* ) and ( *f* ), the bar graphs depicted below the blots represent the CIP activity, calculated as the ratio between the p42/44 (phosphorylated Pmk1) and HA (total Pmk1) signals. They show the mean and standard deviation. The Tukey correction was used after ANOVA in ( *a* ), ( *e* ) and ( *f* ). The Šidák correction was used in ( *g* ). ns, non-significant; * *p* < 0.05. ** *p* < 0.01; *** *p* < 0.001; **** *p* < 0.0001. a.u., arbitrary units. In ( *b– d* ), scale bar, 5 µm.

Exomer collaborates with clathrin adaptors to maintain the integrity of the Golgi and to promote vesicle trafficking to the cell surface and vacuoles [29,76]. We wondered whether the defects in CIP activation were specific to the exomer mutants or a common feature of mutants defective in vesicle trafficking from the TGN. As shown in figure 7*a*, CIP activation by KCl was reduced in both *apm1Δ* (defective in vesicle trafficking to the PM because it lacks in the µ subunit of AP-1 [77]) and *gga21Δ gga22Δ* (defective in vesicle trafficking to the vacuoles because it lacks both GGA adaptors [29,72]). This result shows that vesicle trafficking from the TGN is essential for effective CIP activation in response to osmotic stress.

Next, we compared the distribution of Pck2 in the *apm1Δ* and *gga21Δ gga22Δ* mutants with that of the WT and *cfr1Δ* strains, and found that it was different even in the absence of KCl. To document this observation, we drew line-scans along 10 µm of the cell poles, as indicated in the right panel of figure 7*b*, and plotted the relative fluorescence intensity of each point along the line. The Pck2 distribution was similar in WT and *cfr1Δ* cells (figure 7*b*, left panel). In *apm1Δ* cells, the distribution of Pck2 along the cell poles was similar to that of WT and *cfr1Δ* cells (figure 7*c*). However, in 33.8% of the cells that were undergoing cytokinesis, there was Pck2-GFP fluorescence at the septal area and the cell sides (figure 7*c*, right panel), a distribution that we never observed in WT, *cfr1Δ* or *gga21Δ gga22Δ* cells, which only exhibited Pck2 at the septal area or the poles. In the case of *gga21Δ gga22Δ* cells, the distribution of Pck2 at the cell poles was abnormal, it was off centre (distributed asymmetrically to one side of the pole; cells i and ii in figure 7*d*) or subapical (absent from the centre of the pole; cell iii in figure 7*d*). In summary, each mutant had some defect in the distribution of Pck2. Regarding Pck2 association with the PM, over 90% of the cells from all the strains exhibited Pck2-GFP fluorescence at the cell poles when they grew in the absence of KCl (figure 7*e* and electronic supplementary material, figure S10A). In all the strains, there was a dramatic reduction in this percentage 15 min after the addition of 0.6 M of KCl. Forty minutes after osmotic shock, Pck2 re-localized to the cell poles in about 70% of WT, *apm1Δ* and *gga21Δ gga22Δ* cells, but this phenomenon did not occur in *bch1Δ* cells. For some unknown reason, *apm1Δ* and *gga21Δ gga22Δ* cells exhibited stronger Pck2-GFP fluorescence at 40’ than at 0’ of incubation in KCl (electronic supplementary material, figure S10A).

These results demonstrate that different defects in Pck2 distribution correlate with inefficient CIP activation, and that the defect in Pck2 re-association with the cell surface after osmotic shock is not common to all mutants defective in vesicle traffic from the TGN.

Next, we investigated whether eliminating exomer and a clathrin adaptor would have an additive effect on CIP activation. Because the *cfr1Δ apm1Δ* and *cfr1Δ gga21Δ gga22Δ* strains accumulate multiple defects and grow slowly [29], we addressed this question by analysing CIP in the *ent3Δ* and *cfr1Δ ent3Δ* strains. Ent3 is an epsin that acts in the same functional pathway as the GGAs because it interacts physically with them, depends on them for its localization in the Golgi, and collaborates with them in vesicle trafficking between the TGN and the prevacuolar compartment [72,78]. We found that CIP activation in *ent3Δ* was defective, and that its defect was enhanced in the *cfr1Δ ent3Δ* mutant (figure 7*f*). This result indicates genetic interaction between Cfr1 and clathrin adaptors, and suggests that they act in different pathways for CIP activation.

Finally, we analysed whether *apm1Δ* and/or *gga21Δ gga22Δ* cells accumulated GFP-h\FAPP1(PH) in the TGN. As shown in figure 7*g* and electronic supplementary material, figure S10B, these mutants did not exhibit this phenotype, which was present in *cfr1Δ*.

In summary, these results show that all the mutants defective in vesicle trafficking from the Golgi have a defect in CIP activation, but only exomer mutants are defective in Pck2 re-association with the PM and PI4P accumulation in the TGN.

## 3. Discussion

We have found that in fission yeast, an efficient CIP response to osmotic stress requires exomer. The fact that we observed this defect in response to high concentrations of potassium salts and sorbitol was surprising, because the growth of exomer mutants was defective on plates with 0.6 M of KCl but not 1.2 M of sorbitol [29,32]. This result indicates that other defects in the mutants, such as altered K^+^ homeostasis and abnormal distribution of the K^+^-extruding pump Cta3 [32], might contribute to KCl sensitivity.

In the absence of exomer, defective association of Pck2 with the PM after the initial shock contributes to the inefficient CIP response, and a defect in PI homeostasis is responsible for the reduced association. This finding is consistent with the fact that these lipids regulate the PKCs [62]. The efficiency of the CIP response correlates with the level of Its3 activity [56] (electronic supplementary material, figure S6C), which suggests that the main PI involved is PI(4,5)P2. We have found that bacterially produced Pck2 has strong affinity for cardiolipin (CL) and for phosphatidic acid (PA), and weaker affinity for phosphatidylserine (PS), sulfatide and PI4P. Since CL is present in the inner mitochondrial membrane, and sulfatide is present in the nervous system, their binding to *S. pombe* Pck2 probably lacks biological significance. The analysis of the distribution of PS strongly suggests that a defect in this PL is not responsible for the defect in Pck2 association with the PM. On the contrary, we found that PI4P and PA played a role in the process. PA is a negative molecule with high affinity for positively charged residues, which might explain its strong binding to Pck2. This lipid is the simplest PL, is present in the PM, and serves as the precursor for more-complex PLs such as PIs, PS, phosphatidylethanolamine (PE) and phosphatidylcholine (PC) [68,69] through its conversion to diacylglycerol by the action of lipin phosphatase. Therefore, the suppression of the *cfr1Δ* and *bch1Δ* defects in Pck2 association with the PM by *nem1Δ* might be exerted through PA conversion to PI4P and/or PI(4,5)P2, which would accumulate in the PM.

Although *in vitro* Pck2 binding to PI4P was weak, the role of this lipid in Pck2 stabilization in the PM was demonstrated by increasing the expression of *stt4+*, which suppressed the *cfr1Δ* and *bch1Δ* defect in the association of this protein with the PM. The *P.act1-stt4+* construct produced slightly swollen cells (electronic supplementary material, figure S7A), indicative of PI4P overproduction. However, we did not observe a significant increase in the GFP-2xPH(Osh2) fluorescence. This was probably due to the following non-mutually exclusive reasons: (i) the Stt4 scaffolds Efr3 and Ypp1 [79] were not overexpesssed, which would result in an increase in the amount of PI4P that was limited; and (ii) PI4P was being rapidly converted to PI(4,5)P2 [66]. The *P.act1-stt4+* construct probably ensures a continuous source of PI4P that stabilizes Pck2 in the PM through direct binding, through binding to other protein(s) that stabilize Pck2 and/or by supplying the Its3 substrate.

PIs do not distribute uniformly in all the cell membranes; thus, PI(4,5)P2 is abundant in the PM, and PI4P is present in the PM and abundant in the Golgi. The PM pool of PI4P is the precursor of PI(4,5)P2 and participates in endocytosis, non-vesicular lipid transport, membrane homeostasis and cell signalling pathways, and the Golgi PI4P pool participates in polarized secretion and signalling [71,80–82]. Nevertheless, in mammalian cells, PI4P and PI(4,5)P2 are independent determinants of PM identity, and PI4P contributes to some functions previously attributed specifically to PI(4,5)P2 [83]. The PI4P pools are synthetized *in situ* by specific 1-phosphatidylinositol 4-kinases, and therefore the contribution of the Golgi PI4P to the PM pool is not clear. In mammalian cells, maintenance of the PM PI(4,5)P2 and regulation of ion channels depend on the PM and Golgi pools of PI4P [66]. On the other hand, in budding yeast, recruitment of the Rho1 GEF Rom2 to the PM, and consequently activation of the PKC-mediated MAPK cascade, requires the Stt4-dependent pool of PI(4,5)P2, but not the Pik1-dependent pool [84]. In *S. pombe*, the PI4P generated by Stt4 is the pool that originates the PM PI(4,5)P2 [85]. Here we have shown that in *S. pombe* the PM PI4P pool is not just a precursor of PI(4,5)P2; rather, it is an important determinant for Pck2 association with the PM and CIP signalling. Additionally, the Golgi pool contributes to signalling by replenishing the PM pool after osmotic shock.

Why are exomer mutants only defective in CIP signalling when the osmotic pressure changes? Changes in osmolarity alter the tension and physical properties of membranes. Moreover, membrane lipidomics change in response to osmotic stress in budding yeast and plants [86,87]. We have observed several changes in the distribution of lipids in *S. pombe* treated with high concentrations of KCl; these changes are enhanced in exomer mutants [32] (electronic supplementary material, figures S4 and S6). Thus, it is possible that osmotic stress induces a PM alteration that in exomer mutants is either more deleterious or less efficiently repaired. In *Caenorhabditis elegans* epidermal cells, the Golgi participates in a PM-repairing mechanism that activates in response to damage produced by mechanical stress. This mechanism induces an increase in the arrival of Golgi-derived membranes to the wound, which provides PI4P for enhanced and local generation of PI(4,5)P2 to stabilize the membrane [88]. In *S. pombe*, high KCl concentrations induced a change in the distribution of PI(4,5)P2 [32,56], which was more evident in the absence of exomer, where this lipid was associated into clusters for longer times (electronic supplementary material, figures S4A and 6D). This prolonged clustering might reduce its accessibility. We propose a model (figure 6*f*) in which osmotic shock would result in a reduced amount or accessibility of PI(4,5)P2. Consequently, the cells would require a greater supply of this lipid, which would be obtained from the local phosphorylation of PI4P by Its3. This acute response would result in a high demand of PI4P, and in a shortage of this lipid for other functions. Under these circumstances, Golgi PI4P, synthesized by Pik1, would be delivered to the PM through an exomer-facilitated mechanism. The enhanced PI4P supply would also serve as an anchor for Pck2, which would re-associate with the PM and would be available for new rounds of CIP activation when required.

Exomer is a protein complex with vesicle coat and cargo adaptor properties that binds lipids and proteins, and facilitates the transport of trans-membrane proteins that localize to the sites of polarized growth [11–13,15–19,22,23,25,89]. Here we have found that *S. pombe* exomer regulates Pck2, Rgf1 and Mkh1, which are not trans-membrane proteins but associate with lipids and exhibit a polarized distribution. We have also found that correct lipid distribution, in particular in response to osmotic stress, depends on exomer. More specifically, we found a defect in PI4P homeostasis in exomer mutants. The mechanism by which exomer could modulate PI4P transport remains unknown and requires further research. Exomer interacts with lipids and Arf1 [11–13,15,16,23]. Therefore, it might be involved in lipid partitioning during vesicle budding [90,91] and/or in modulating Arf1. Clathrin adaptors that operate in the Golgi also interact with lipids and Arf1, and some results suggest that they might collaborate or compete with exomer [29,76]. Our results show that these adaptors also regulate the CIP in response to osmotic stress and, according to the genetic interaction between *ent3Δ* and *cfr1Δ*, the more defective the trafficking from the TGN, the less effective is CIP activation. This view is consistent with the role of the Golgi in signalling [40,41]. Nevertheless, this genetic interaction, and the fact that cells lacking these adaptors are not defective in Pck2 re-association with the PM and do not accumulate PI4P in the Golgi, strongly suggest that exomer and clathrin adaptors act through a different mechanism, and that regulating PI4P transport from the Golgi might be a specific exomer function.

Exomer mutants exhibit mild defects in multiple processes: signalling in response to osmotic stress, cell wall synthesis, cytokinesis, mating, PM ATPase activity, polarized secretion, potassium and calcium homeostasis, and lipid distribution (data from this study and [17–19,24,29,31,32]). Since the lipid environment influences protein distribution and enzyme activity, it is possible that all those defects are the consequence of altered lipid trafficking in the absence of exomer. Accordingly, the function of exomer should be contemplated from not only a protein-centric perspective, but also a lipid-centric perspective.

## 4. Material and methods

### 4.1. Strains and growth conditions

The yeast strains used in this work are listed in electronic supplementary material, table S1. We have followed the nomenclature suggested by Lera-Ramirez *e* *t al.* [92] . The *cfr1Δ* and *bch1Δ* mutants, which have the same phenotypes ([29] and results not shown) were used indistinctly as exomer mutants. YES medium (0.5 % yeast extract, 3 % glucose, 225 mg l ^−^^1^ adenine, histidine, leucine, uracil and lysine hydrochloride, 2 % agar, pH 5.6) was used for the growth and maintenance of *S. pombe* strains, as described previously [93,94]. When required, geneticin (G418, Formedium), hygromycin B (Formedium) and nourseothricin (Werner BioAgents) were used at 120, 200 and 50 μg ml ^−^^1^ , respectively. Drop-test analyses were performed as described previously [29]. Samples for biochemical and microscopy analyses were taken from cultures growing exponentially in YES liquid medium at 28 °C. To induce a stress response, samples were collected at various times after adding different compounds (KCl and CaCl_2_ from Sigma, KNO_3_ from Merck, C_2_H_3_KO_2_ from Panreac or caspofungin from MSD) to the cultures, or after incubation at 40 °C in an orbital shaker. To analyse the response to sorbitol-induced hyperosmotic stress, cells were collected by filtration, and the filters were introduced into flasks with YES medium supplemented with 1.2 M sorbitol (Sigma), and incubated. For hypotonic stress, cells growing in YES medium with 0.8 M sorbitol were collected by filtration, introduced into flasks with YES medium and incubated.

### 4.2. Genetic methods

Molecular and genetic manipulations were performed according to Sambrook *et al* . [95]. Gene deletions and C-terminally tagged proteins were generated by transforming the strain of interest with PCR-generated modules, as described previously [96]. We used the strong constitutive promoter *P.act1+* to enhance the expression of several genes. Although this strategy might result in a weaker overexpression than that produced by the pREp3X plasmids, we preferred it because it allowed an enhanced expression without nutritional changes. The *P.act1-pek1DD*-T*nda2* construct was generated as follows (see electronic supplementary material). A 1 kb *Pst*I/*Apa*I DNA fragment upstream of the *act1+* open reading frame was amplified by PCR, and ligated to the *pek1DD* mutant open-reading frame (ORF) (purchased as an *Apa*I/*Sal*I synthetic gene from Integrated DNA Technologies (IDT) , USA) into a pINTH-Stx8 plasmid [97,98] digested with *Pst*I and *Sal*I. In this way, *pek1DD* was under the control of the strong actin promoter (P.*act1+*), and the *nda2+* terminator. The sequence of interest was liberated by digestion with *Not*I, and integrated into the exogenous *hph.171K* locus [97]. To express the 1-phosphatidylinositol 4-phosphate 5-kinase *its3+* from the *act1+* promoter, a synthetic sequence that included *its 3+* 5′ untranslated (UTR) sequences, the *HPHMX6* hygromycin resistance gene, a 1 kb DNA region upstream of the *act1+* ORF and the initial 218 bp of the *its3+* ORF was purchased from IDT and then cloned into the pUC19 vector. A similar approach was used to express the 1-phosphatidylinositol 4-kinases *stt4+* and *pik1+* genes from the *act1+* promoter. The sequences of interest are described in the electronic supplementary material. The DNA fragments were liberated by enzyme digestion and used to transform yeast cells. The accuracy of the constructs and integrations were assessed by DNA sequencing and PCR, respectively. Genetic crosses and selection of the characters of interest by random spore analysis [93] were used to combine different traits.

### 4.3. Protein methods

To assess the level of different GFP-tagged proteins, trichloroacetic acid (TCA) protein precipitation from cell extracts and western blot analyses were performed as described previously [72]. Cells growing exponentially in 30 ml of YES were collected by centrifugation (900 × *g* ), washed with 1 ml of cold 20 % TCA, and resuspended in 50 µl of the same solution. Five hundred microlitres of glass beads (Braun Biotech International) was added and the cells were broken in a cold Fast Prep FP120 (Savant Bio101) using three 16 s pulses (speed 6), with 5 min incubations on ice between pulses. Four hundred microlitres of cold 5 % TCA was added to the tube, which was vortexed to wash the beads. Cell extracts were transferred to a clean tube and centrifuged for 10 min at 4 °C. The pellets were resuspended in 2 % sodium dodecyl sulfate (SDS)/0.3 M Tris Base. The protein concentration was determined using the Bradford protein assay reagent (Bio-Rad). Equal amounts of protein were boiled in the presence of Laemmli sample buffer (50 mM Tris-HCl ( pH 6.8) , 1 % SDS, 143 mM β-mercaptoethanol, 10 % glycerol) for 5 min . The samples were subjected to polyacrylamide gel electrophoresis (PAGE), transferred to polyvinylidene difluoride (PVDF) membranes and incubated with non-fat dried milk to block nonspecific protein binding (Nestlé; 5 % in TBST: 0.25 % Tris-HCl, 0.9 % NaCl, 0.25 % Tween 20, pH 6.8). The primary antibodies were anti-GFP (JL8, BD Living Colors; 1:3000) and anti-α tubulin (clone B -5 -1 -2; Sigma, 1:10000). The secondary antibody was horseradish peroxidase (HRP)-conjugated anti-mouse IgG (Bio-Rad no. 170 -6515, 1:10000). The molecular weight standard was PageRuler Plus Prestained Protein Ladder (Thermo Scientific no. 26620). Chemiluminescent signal was generated using the Western Bright ECL detection kit (Advansta no. K -12045-D20), and detected on a Vilber Fusion FX system (Vilber GmbH), which captures chemiluminescence until the best-fitting signal is recorded.

For CIP activation, exponentially growing cells carrying a HA:6His-tagged chromosomal version of *pmk1+* were grown in YES medium to the mid-log phase, treated with the corresponding stressor and incubated at 28 °C for different times. The cells were collected by filtration to avoid stimulation by the shear forces generated during centrifugation [99]. Purification and detection of total and activated Pmk1 was performed as described previously [60], with some modifications. Pmk1-HA:6His was purified using Ni-NTA His-bind resin from Millipore. Non-fat dry milk was used to block nonspecific protein binding on the membrane and to dilute the anti-HA antibody (used to detect total Pmk1), while 3 % bovine serum albumin (BSA; Sigma no. A -9647) was used for blocking and diluting the antibody used to detect activated Pmk1 (anti-phospho-p44/42 MAPK [Erk1/2] [Thr202/Tyr204] antibody, Cell signalling Technology no. 9101 S). The blots were developed as described above. The level of activation of the stress-activated protein kinase pathway was performed with a similar experimental procedure, but the strains had Sty1-HA:6His, and the anti-phospho-p38 MAPK (pTyr322) antibody (Cell Signalling Technology no. 9211) was used to detect Sty1 phosphorylation. To perform protein–lipid overlay assays, GST-Pck2 was purified as described previously [100], with modifications. Briefly, GST-Pck2 was expressed from the pGEX -2T-TEV plasmid in the *Escherichia coli* BL21 strain, which was incubated at 28 °C for 16 h in the presence of isopropyl-β- D -thiogalactopyranoside (IPTG; Merck no. 10724815001). The protein was purified with glutathione-Sepharose 4B beads (Cytiva no. 17075601) and quantified. A portion of the protein (2.5 µg) was used to bind lipids spotted on Membrane Lipid Strips (Echelon no. P - 6002), which was decorated with anti-GST HRP-conjugated antibody (Cytiva no. RPN1236) and developed as described above.

### 4.4. Microscopy

For most of the experiments involving confocal live-cell imaging, a NIKON Ti2-E confocal ‘spinning disk ’ microscope (100×/1.45 Oil Plan Apo objective), equipped with an Andor Dragonfly system and a sCMOS Sona 4.2B -11 camera, was used. Images were processed using deconvolution in the Fusion software from Andor. For the experiments shown in figure 3, 4*c*, 5*a*, 6*c*, 6*d* and 7*e*, the images were captured with an Olympus IX71 microscope (100 × objective; numerical aperture 1.4) equipped with a personal DeltaVision system and a Photometrics CoolSnap HQ2 monochrome camera. In this case, images were processed using deconvolution in the softWoRX DV software from Applied Precision. In both cases, stacks of three Z-series sections that corresponded to the cell middle were acquired at 0.2 μm intervals. The images are average (AVG) projections, which were used instead of SUM projections in order to avoid image reescalation. A GFP-h\FAPP1(PH) probe was used to estimate the presence of PI4P in the TGN [29,72,73]. GFP -2xPH(Osh2) [65] and GFP-h\GAP1IP4BP(PH) [32,101], expressed from the *P.nda2+* promoter were the probes used to detect PI4P and PI(4,5)P2 in the PM, respectively. LactC2-GFP [67,102] was used as a probe to detect PS in the inner PM leaflet. Typically, samples were collected by centrifugation (1 min at 3000 rpm), spotted on a slide and photographed; when images had to be captured at short times after osmotic shock, the cells were fixed with lectin in a µ-Slide VI 0.5 Glass Bottom chamber (ibidi no. 80607); the channel was filled with KCl-supplemented YES medium, and images were captured along the experiment.

To determine the fluorescence intensity of proteins associated with the PM, line-scans were performed by drawing a line across the cell surface and analy sing the maximum fluorescence intensity along the line. We only analysed the maximum fluorescence to reduce the background noise. To estimate the percentage of cells in which Pck2-GFP relocalized to the cell surface after osmotic shock, the Fiji Cell Counter plug-in (ImageJ software, National Institutes of Health) was used to count cells with fluorescent signal. We observed that, after the shock, some cells exhibited fluorescence at the cell sides instead of the poles. We scored those cells that exhibited the signal at the poles and at the cell sides, but not those that exhibited the signal at cell equator (defined by the position of the nuclei, electronic supplementary material, figure S3) and the septa. To determine Pck2-GFP distribution along the cell poles of different strains (figure 7) ), the fluorescence intensity was estimated by drawing a 10 µm line along the poles, and the mean and standard deviation of the relative fluorescence intensity values at each point along the line, with respect to the maximum value, was represented in a graph. For each experiment, a minimum of 20 poles were analysed. To estimate the fluorescence intensity of intracellular structures, the ImageJ Particle Analysis tool was used after defining a threshold. For co-localization, stacks of three 0.20 µm Z-sections of the cell middle were acquired and the central plane of the stacks from both channels were merged.

### 4.5. Statistical analyses

The data that were subjected to quantification were the result of a minimum of three experiments, unless stated otherwise. For microscopy data, a minimum of 120 cells/particles from three different images were scored in each experiment. To calculate the level of CIP activation in a sample, the intensity of phosphorylated Pmk1 (p42/44) and total Pmk1 (HA) bands in the western blot was estimated using Fiji. The ratio between the p42/44 and HA band intensity was calculated. The mean, standard deviation and statistical significance of the differences are depicted in bar graphs below the blot images in each figure. All the data were initially analysed with Excel, and then visualized and statistically analysed with GraphPad Prism. In each case, we used the correction test suggested by GraphPad Prism to determine the statistical significance of the differences.

## Data Availability

The list of strains, DNA sequences of interest, original Westerns and supplemental figures are included in the electronic supplementary material [103].
